# Supplementary material for: Variant landscape of the RYR1 gene based on whole genome sequencing of the Singaporean population
Source: Sci Rep. 2022 Mar 31;12:5429. doi: 10.1038/s41598-022-09310-w (PMC8971428; doi:10.1038/s41598-022-09310-w)
Supplement: Supplementary file 1 — Supplementary Information. [file 41598_2022_9310_MOESM1_ESM.docx]

**List of supplemental material**

Supplementary figure S1: Ethnic distribution of all pathogenic *RYR1* variants

Supplementary figure S2: Ethnic distribution of all pathogenic and likely pathogenic *RYR1* variants

Supplementary figure S3: Ethnic distribution of individual pathogenic and likely pathogenic *RYR1* variants

Supplementary table S1: Clinical significance of *RYR1* variants based on ClinVar

Supplementary table S2: Clinical significance of *RYR1* variants based on InterVar

Supplementary table S3: Diseases associated with pathogenic and likely pathogenic RYR1 variants with supporting evidence from ClinVar

Supplementary table S4: Conservation of mutation sites. Evidence was obtained from Clinvar

Supplementary table S5: Allele frequencies of the pathogenic and likely pathogenic *RYR1* variants

Supplementary table S6: Allele frequency comparisons of pathogenic and likely pathogenic *RYR1* variants between different populations

Supplementary figure S1: Ethnic distribution of all pathogenic *RYR1* variants

Supplementary figure S2: Ethnic distribution of all pathogenic and likely pathogenic *RYR1* variants

Supplementary figure S3: Ethnic distribution of individual pathogenic and likely pathogenic *RYR1* variants

Supplementary table S1: Clinical significance of *RYR1* variants based on ClinVar.

| Clinvar classification | Number (percentage) of *RYR1* variants |
| --- | --- |
| Pathogenic | 4 (0.7%) |
| Likely Pathogenic | 2 (0.3%) |
| Variant of uncertain significance | 88 (15.6%) |
| Likely benign | 0 (0.0%) |
| Benign | 0 (0.0%) |
| Conflicting* | 33 (5.9%) |
| Unclassified | 437 (77.5%) |

*Conflicting refers to when different submitters assign different clinical significance to a variant. This includes variants which have the following combination of classifications from different submitters:

1. (Pathogenic or Likely pathogenic or Benign or Likely benign) AND Uncertain significance

2. (Pathogenic or Likely pathogenic) AND (Benign or Likely benign)

Supplementary table S2: Clinical significance of *RYR1* variants based on InterVar.

| Intervar classification | Number (percentage) of *RYR1* variants |
| --- | --- |
| Pathogenic | 1 (0.2%) |
| Likely Pathogenic | 6 (1.1%) |
| Variant of uncertain significance | 274 (48.5%) |
| Likely benign | 237 (42.0%) |
| Benign | 28 (5.0%) |
| Unclassified | 18 (3.2%) |

Supplementary table S3: Diseases associated with pathogenic and likely pathogenic RYR1 variants with supporting evidence from ClinVar

| **Variant** | **Interpreted conditions** | **ClinVar interpretation** | **Evidence** |
| --- | --- | --- | --- |
| c.325C>T (p.Arg109Trp)  *rs118192173* | Minicore myopathy with external opthalmoplegia | Pathogenic | Mutation found in 2 minicore myopathy patients from 1 family with disease onset in the neonatal period (<https://pubmed.ncbi.nlm.nih.gov/16380615/>) |
|  | Congenital myopathy with fibre type disproportion | Likely pathogenic | The following ACMG criteria were applied in classifying this variant: PS1, PM2, PP3*. |
| c.6487C>T (p.Arg2163Cys)  *rs118192175* | 1. Malignant hyperthermia susceptibility  2. Central core myopathy | Pathogenic | 1. Mutation found in individuals from 2 families who had malignant hyperthermia episodes ([https://www.cell.com/ ajhg/fulltext/S0002-9297(07)63840-3](https://www.cell.com/%20ajhg/fulltext/S0002-9297(07)63840-3)); annotated in PharmGKB database as associated with Malignant Hyperthermia (Level 1A evidence) (<https://www.pharmgkb.org/clinicalAnnotation/1183705797>)  2. Submitted from GeneReviews, converted during submission to pathogenic (<https://www.ncbi.nlm.nih.gov/clinvar/submitters/500062/>) |
| c.7268T>A (p.Met2423Lys)  *rs118192174* | 1. Minicore myopathy with external ophthalmoplegia  2. Congenital myopathy with fibre type disproportion  3. Clubfoot  4. EMG abnormality  5. Lower limb amyotrophy | Pathogenic | 1. Mutation found in 4 individuals from 1 family with disease onset in infancy and childhood (<https://pubmed.ncbi.nlm.nih.gov/16380615/>)  2-5. Submitted by Centre for Mendelian Genomics, University Medical Centre Ljubljana (<https://www.ncbi.nlm.nih.gov/clinvar/submitters/505952/>) |
| c.14111C>T (p.Thr4704Met)  *rs118192140* | 1. Minicore myopathy with external ophthalmoplegia  2. Central core myopathy | Pathogenic | 1. Mutation associated with clinical features of the disease and molecular studies showed an association with lower levels of RYR1 protein on western blot analysis (<https://pubmed.ncbi.nlm.nih.gov/17483490/>)  2. Submitted from GeneReviews (  <https://www.ncbi.nlm.nih.gov/clinvar/variation/65996/evidence/>) |
|  | 1. Central core myopathy  2. Congenital myopathy with fiber type disproportion  3. Malignant hyperthermia susceptibility  4. Minicore myopathy with external ophthalmoplegia | Likely pathogenic | Submitted from Fulgent Genetics (<https://www.ncbi.nlm.nih.gov/clinvar/variation/65996/evidence/>) |
|  | Malignant hyperthermia susceptibility | Variant of uncertain significance | This variant was observed as part of a predisposition screen in an ostensibly healthy population. A literature search was performed for the gene, cDNA change, and amino acid change (where applicable). No publications were found based on this search. Allele frequency data from public databases did not allow this variant to be ruled in or out of causing disease. Therefore, this variant is classified as a variant of unknown significance. (<https://www.ncbi.nlm.nih.gov/clinvar/variation/65996/evidence/>) |
| c.742G>T (p.Gly248Trp)  *rs1801086* | Malignant hyperthermia | Likely pathogenic | Submitted by pharmGKB (https://www.ncbi.nlm.nih.gov/clinvar/RCV000786702/) |
| c.1186G>T (p.Glu396X)**  *rs774919231* | NA | NA | NA |
| c.2654G>A (p.Arg885His)  *rs370634440* | 1. Malignant hyperthermia susceptibility  2. Minicore myopathy with external ophthalmoplegia  3. Congenital myopathy with fiber type disproportion | Uncertain significance | 1. This variant was observed in the ICSL laboratory as part of a predisposition screen in an ostensibly healthy population. It had not been previously curated by ICSL or reported in the Human Gene Mutation Database (HGMD: prior to June 1st, 2018), and was therefore a candidate for classification through an automated scoring system. Utilizing variant allele frequency, disease prevalence and penetrance estimates, and inheritance mode, an automated score was calculated to assess if this variant is too frequent to cause the disease. Based on the score, this variant could not be ruled out of causing disease and therefore its association with disease required further investigation. A literature search was performed for the gene, cDNA change, and amino acid change (if applicable). No publications were found based on this search. This variant was therefore classified as a variant of unknown significance for this disease. (<https://www.ncbi.nlm.nih.gov/clinvar/variation/212100/?new_evidence=false)>  2, 3. Submitted from Genomic Research Center, Shahid Beheshti University of Medical Services and Illumina Clinical Services Laboratory |
| c.8554C>T (p.Arg2852X)  *rs886054396* | RYR1-related disorders | Variant of uncertain significance | The RYR1 c.8554C>T (p.Arg2852Ter) stop-gained variant has been reported in one study in which it was found in a compound heterozygous state in one individual with muscular dystrophy and arthrogryposis (Vasli et al. 2012). The individual's affected twin brother was a compound heterozygote for the same two variants, but it is not known whether the twin brothers were monozygotic or dizygotic. The p.Arg2852Ter variant was also found in a heterozygous state in an unaffected sibling and an unaffected parent. Control data are unavailable for this variant, and the variant is not found in the 1000 Genomes Project, the Exome Sequencing Project, or the Exome Aggregation Consortium. The disease description in this family is most consistent with multiminicore disease, but there is considerable overlap of disease symptoms with central core disease and congenital neuromuscular disease with uniform type 1 fiber. **The p.Arg2852Ter variant has not been reported in the literature in association with malignant hyperthermia susceptibility**. Due to the potential impact of stop-gained variants and the evidence from the literature, the p.Arg2852Ter variant is classified as a variant of unknown significance but suspicious for pathogenicity for RYR1-related disorders. This variant was observed by ICSL as part of a predisposition screen in an ostensibly healthy population. (<https://www.ncbi.nlm.nih.gov/clinvar/variation/329081/?new_evidence=false)> |

*PS1: same amino acid change known; PM2: absent from controls; PP3: in silico evidence

**Not found in ClinVar. Labelled as pathogenic in InterVar.

Supplementary table S4: Conservation of mutation sites. Evidence was obtained from Clinvar.

| Variant | Clinical significance | Evidence |
| --- | --- | --- |
| c.325C>T (p.Arg109Trp) | Pathogenic | This sequence change replaces arginine with tryptophan at codon 109 of the *RYR1* protein (p.Arg109Trp). The arginine residue is highly conserved and there is a moderate physicochemical difference between arginine and tryptophan. |
| c.6487C>T (p.Arg2163Cys) | Pathogenic | The R2163C variant is a non-conservative amino acid substitution, which occurs at a position that is conserved across species. |
| c.7268T>A (p.Met2423Lys) | Pathogenic | In 3 sibs with minicore myopathy with external ophthalmoplegia (255320) originally reported by Swash and Schwartz (1981), Jungbluth et al. (2005) identified a 7268T-A transversion in exon 45 the *RYR1* gene, resulting in a met2423-to-lys substitution in a highly conserved region. |
| c.14111C>T (p.Thr4704Met) | Pathogenic | This sequence change replaces threonine with methionine at codon 4709 of the *RYR1* protein (p.Thr4709Met). The threonine residue is highly conserved and there is a moderate physicochemical difference between threonine and methionine. |
| c.742G>T (p.Gly248Trp) | Likely pathogenic | No evidence about the conservation of the mutation site. |
| c.1186G>T (p.Glu396X) | Likely pathogenic | No evidence about the conservation of the mutation site. |
| c.2654G>A (p.Arg885His) | Likely pathogenic | This sequence change replaces arginine with histidine at codon 885 of the *RYR1* protein (p.Arg885His). The arginine residue is highly conserved and there is a small physicochemical difference between arginine and histidine. |
| c.8554C>T (p.Arg2852X) | Likely pathogenic | No evidence about the conservation of the mutation site. |

Supplementary table S5: Allele frequencies of the pathogenic and likely pathogenic *RYR1* variants

| **Variant** | **All_AF (%)** | **CHS_AF (%)** | **INS_AF (%)** | **MAS_AF (%)** | **ExAC_ALL (%)** | **GnomAD_exome_ALL (%)** | **AMR_AF (%)** | **NFE_AF(%)** | **SAS_AF (%)** | **SEA_AF (%)** | **NEA_AF (%)** |
| --- | --- | --- | --- | --- | --- | --- | --- | --- | --- | --- | --- |
| c.325C>T | 0.01 | 0.018 | 0 | 0 | 0.01 | 0.008 | 0.009 (ExAC)  0.003 (gnomAD) | 0.02 (ExAC)  0 (gnomAD) | 0.006 (ExAC)  0 (gnomAD) | Nil | Nil |
| c.6487C>T | 0.01 | 0 | 0 | 0.055 | Nil | 0.001 | 0.003 (gnomAD) | 0 (gnomAD) | 0.02 (gnomAD) | Nil | Nil |
| c.7268T>A | 0.03 | 0 | 0.133 | 0 | 0.004 | 0.002 | 0 (ExAC)  0.02 (gnomAD) | 0 (ExAC and gnomAD) | 0.03 (ExAC)    0 (gnomAD) | Nil | Nil |
| c.14111C>T | 0.01 | 0 | 0.044 | 0 | 0.002 | 0.004 | 0 (ExAC)  0.003 (gnomAD) | 0.003 (ExAC)  0 (gnomAD) | 0 (ExAC and gnomAD) | Nil | Nil |
| c.742G>T | 0.01 | 0.018 | 0 | 0 | 0.001 | 0.001 | 0 (ExAC and gnomAD) | 0 (ExAC and gnomAD) | 0 (ExAC and gnomAD)  0.069 (genomeasia 100k) | 0 (genomeasia 100K) | 0 (genomeasia 100k) |
| c.1186G>T | 0.03 | 0 | 0 | 0.166 | 0.001 | 0.001 | 0 (ExAC and gnomAD) | 0.002 (ExAC)  0 (gnomAD) | 0 (ExAC and gnomAD) | Nil | Nil |
| c.2654G>A | 0.031 | 0 | 0.133 | 0 | 0.02 | 0.02 | 0 (ExAC)  0.04 (gnomAD) | 0.02 (ExAC)  0 (gnomAD) | 0.04 (ExAC)  0 (gnomAD) | Nil | Nil |
| c.8554C>T | 0.01 | 0.018 | 0 | 0 | Nil | 0 | 0 (gnomAD) | 0 (gnomAD) | 0 (gnomAD) | Nil | Nil |

Orange: defined as pathogenic in this study; Blue: defined as likely pathogenic in this study.

**Abbreviations**: All_AF: allele frequency in the Singapore population; CHS_AF: allele frequency amongst Chinese; INS: allele frequency amongst Indians; MAS: allele frequency amongst Malays; ExAC_ALL: allele frequency in the ExAC browser; GnomAD_exome ALL: allele frequency in the gnomAD browser; AMR_AF: allele frequency in the American population; NFE_AF: allele frequency in the Non-Finnish European population; SAS_AF: allele frequency in the South Asian population; SEA_AF: allele frequency in the Southeast Asian population; NEA_AF: allele frequency in the Northeast Asian population. Nil: no data available.

**Subject breakdown in various databases**: ExAC: ALL 60,706, AMR 5,789, NFE 33,370, SAS 8,256; GnomAD: ALL 141,456, AMR 17,720, NFE 64,603, SAS 15,308; GenomeAsia 100K: NEA 351, SAS 724, SEA 346

Supplementary table S6: Allele frequency comparisons of pathogenic and likely pathogenic *RYR1* variants between different populations

| **Variant** | ***P* ALL (SG5K) vs ALL (ExAC)** | ***P* ALL (SG5K) vs ALL (GnomAD)** | ***P* ALL (SG5K) vs AMR (ExAC)** | ***P* ALL (SG5K) vs AMR (GnomAD)** | ***P* ALL (SG5K) vs NFE (ExAC)** | ***P* ALL (SG5K) vs NFE (GnomAD)** | ***P* ALL (SG5K) vs SAS (ExAC)** | ***P* ALL (SG5K) vs SAS (GnomAD)** | ***P* ALL (SG5K) vs SAS (genomeasia 100k)** | ***P* ALL (SG5K) vs SEA (100K)** | ***P* ALL (SG5K) vs NEA (100K)** |
| --- | --- | --- | --- | --- | --- | --- | --- | --- | --- | --- | --- |
| c.325C>T | 0.589 | 0.407 | 0.5 | 0.046 | 0.048 | 9.54×10^-4^ | 0.226 | 9.54×10^-4^ | Nil | Nil | Nil |
| c.6487C>T | Nil | 0.006 | Nil | 0.046 | Nil | 9.54×10^-4^ | Nil | 0.048 | Nil | Nil | Nil |
| c.7268T>A | 2.61×10^-6^ | 1.02×10^-7^ | 7.47×10^-10^ | 0.098 | 7.47×10^-10^ | 7.47×10^-10^ | 0.552 | 7.47×10^-10^ | Nil | Nil | Nil |
| c.14111C>T | 0.019 | 0.089 | 9.54×10^-4^ | 0.046 | 0.046 | 9.54×10^-4^ | 9.54×10^-4^ | 9.54×10^-4^ | Nil | Nil | Nil |
| c.742G>T | 0.006 | 0.006 | 9.54×10^-4^ | 9.54×10^-4^ | 9.54×10^-4^ | 9.54×10^-4^ | 9.54×10^-4^ | 9.54×10^-4^ | 1.13×10^-12^ | 9.54×10^-4^ | 9.54×10^-4^ |
| c.1186G>T | 1.21×10^-8^ | 1.21×10^-8^ | 7.47×10^-10^ | 7.47×10^-10^ | 1.02×10^-7^ | 7.47×10^-10^ | 7.47×10^-10^ | 7.47×10^-10^ | Nil | Nil | Nil |
| c.2654G>A | 0.077 | 0.077 | 3.68×10^-10^ | 0.167 | 0.078 | 3.68×10^-10^ | 0.887 | 3.68×10^-10^ | Nil | Nil | Nil |
| c.8554C>T | Nil | 9.54×10^-4^ | Nil | 9.54×10^-4^ | Nil | 9.54×10^-4^ | Nil | 9.54×10^-4^ | Nil | Nil | Nil |

Orange: defined as pathogenic in this study; Blue: defined as likely pathogenic in this study; Green: defined as AF in SG5K significantly higher than other population; Red: AF in SG5K significantly lower than other population.

Abbreviations: All_AF: allele frequency in the Singapore population; CHS_AF: allele frequency amongst Chinese; INS: allele frequency amongst Indians; MAS: allele frequency amongst Malays; ExAC_ALL: allele frequency in the ExAC browser; GnomAD_exome ALL: allele frequency in the gnomAD browser; AMR_AF: allele frequency in the American population; NFE_AF: allele frequency in the Non-Finnish European population; SAS_AF: allele frequency in the South Asian population; SEA_AF: allele frequency in the Southeast Asian population; NEA_AF: allele frequency in the Northeast Asian population. Nil: no data available
